# MR Spectroscopy of the Insula: Within- and between-Session Reproducibility of MEGA-PRESS Measurements of GABA+ and Other Metabolites

**DOI:** 10.3390/brainsci11111538

**Published:** 2021-11-19

**Authors:** Claire Shyu, Sonja Elsaid, Peter Truong, Sofia Chavez, Bernard Le Foll

**Affiliations:** 1 Translational Addiction Research Laboratory, Centre for Addiction and Mental Health, University of Toronto, Toronto, ON M5S 2S1, Canada; claire.shyu@camh.ca (C.S.); sonja.elsaid@camh.ca (S.E.); 2 Brain Health Imaging Centre, Centre for Addiction and Mental Health, Toronto, ON M5T 1R8, Canada; peter.truong@sri.utoronto.ca (P.T.); sofia.chavez@utoronto.ca (S.C.); 3 Department of Pharmacology and Toxicology, University of Toronto, Toronto, ON M5S 1A8, Canada; 4 Institute of Medical Sciences, University of Toronto, Toronto, ON M5S 1A8, Canada; 5 Campbell Family Mental Health Research Institute, Centre for Addiction and Mental Health, Toronto, ON M5T 1R8, Canada; 6 Department of Psychiatry, Division of Brain and Therapeutics, University of Toronto, Toronto, ON M5T 1R8, Canada; 7 Concurrent Outpatient Medical & Psychosocial Addiction Support Services, Centre for Addiction and Mental Health, Toronto, ON M6J 1H4, Canada; 8 Acute Care Program, Centre for Addiction and Mental Health, Toronto, ON M6J 1H3, Canada; 9 Department of Family and Community Medicine, University of Toronto, Toronto, ON M5T 1R8, Canada

**Keywords:** insula, gamma-aminobutyric acid, GABA, LCModel, MEGA-PRESS, PRESS, magnetic resonance spectroscopy, MRS, reproducibility

## Abstract

The insula plays a critical role in many neuropsychological disorders. Research investigating its neurochemistry with magnetic resonance spectroscopy (MRS) has been limited compared with cortical regions. Here, we investigate the within-session and between-session reproducibility of metabolite measurements in the insula on a 3T scanner. We measure N-acetylaspartate + N-acetylaspartylglutamate (tNAA), creatine + phosphocreatine (tCr), glycerophosphocholine + phosphocholine (tCho), myo-inositol (Ins), glutamate + glutamine (Glx), and γ-aminobutyric acid (GABA) in one cohort using a j-edited MEGA-PRESS sequence. We measure tNAA, tCr, tCho, Ins, and Glx in another cohort with a standard short-TE PRESS sequence as a reference for the reproducibility metrics. All participants were scanned 4 times identically: 2 back-to-back scans each day, on 2 days. Preprocessing was done using LCModel and Gannet. Reproducibility was determined using Pearson’s *r*, intraclass-correlation coefficients (ICC), coefficients of variation (CV%), and Bland–Altman plots. A MEGA-PRESS protocol requiring averaged results over two 6:45-min scans yielded reproducible GABA measurements (CV% = 7.15%). This averaging also yielded reproducibility metrics comparable to those from PRESS for the other metabolites. Voxel placement inconsistencies did not affect reproducibility, and no sex differences were found. The data suggest that MEGA-PRESS can reliably measure standard metabolites and GABA in the insula.

## 1. Introduction

The insula, a bilateral region of the cerebral cortex, plays a major role in human cognition, interoception, sensorimotor, and socio-emotional processing [1]. Due to its location deep within the lateral sulcus, the insula had largely been overlooked until recent decades, where technological advances provide new insights on its contribution to neuropathologies. Clinical studies have demonstrated its implication in neuropsychiatric disorders, including addiction, schizophrenia, mood, panic, post-traumatic stress, and obsessive-compulsive disorders [2,3,4,5,6,7,8,9]. The insula has also become a subject of interest in addiction research following the discovery that insular damage facilitated smoking cessations [10,11].

One approach to studying the insula involves understanding its neurochemistry. Magnetic Resonance Spectroscopy (MRS) allows non-invasive in-vivo metabolite measurements of a particular brain region. Using MRS, altered metabolite concentrations in the insula were reported in fibromyalgia [12], epilepsy [13,14,15], schizophrenia [16], anxiety disorders [17], major depression [18], and drug addiction [8]. 

MRS uses magnetic fields to excite a localized region of the brain and specialized head coils to receive the signal from the nucleus of interest at a given time referred to as the echo time (TE). For 1H-MRS, the hydrogen atoms in the various metabolites of interest produce a particular molecule-specific signature, for the given TE. The collected spectrum is a superposition of all the signatures of ^1^H-containing molecules (including water). LCModel [19] software uses a linear combination of reference metabolite signatures, collected or simulated, to fit the spectrum produced using a standard MRS technique, such as PRESS (Point RESolved Spectroscopy). The commonly measured metabolites using this method, at a short TE (e.g., TE = 35 ms), are: N-acetylaspartate + N-acetylaspartylglutamate (NAA + NAAG), that will be referred to as total NAA (tNAA); creatine + phosphocreatine (Cr + PCr), that will be referred to as total Cr (tCr); glutamate + glutamine, commonly referred to as Glx (Glx = Glu + Gln); glycerophosphocholine + phosphocholine (GPC + PCh), that will be referred to as total Cho (tCh), and myo-inositol (Ins). We will refer to these five metabolites tNAA, tCr, Glx, tCh, and Ins as the *standard* metabolites because they can be measured using standard MRS sequences (such as PRESS). 

More recently, γ-aminobutyric acid (GABA), a primary inhibitory metabolite in the central nervous system, has become a focus of interest in many neuropsychiatric studies. Levels of GABA have been shown to be altered in the insula for various diseased states, including PTSD [2] and chronic pain [2,20,21]. Due to the low concentration and somewhat spread-out signature of GABA with multiple peaks coinciding with those of other metabolites, advanced specialized MRS sequences have been required to enable GABA measurements. The MEGA-PRESS (MEscher- GArwood Point RESolved Spectroscopy) sequence uses editing pulses to specifically alter the signature of GABA. By acquiring alternating edit-ON and edit-OFF lines of data, averaging and then subtracting these we can produce the difference spectrum (MEGA-PRESS_diff_) [22]. The sought GABA peak can be revealed and quantified by specialized software [23]. Furthermore, the data from the edit-OFF lines can be collected and processed as a regular PRESS sequence with LCModel, albeit with a longer TE. We will refer to this spectrum as the MEGA-PRESS_editOFF_ spectrum to be distinguished from the MEGA-PRESS_diff_ spectrum.

For any methodology, such as MRS, various factors, internal (e.g., physiology and motion) and external (e.g., scanner stability), can have detrimental impacts on the ability to make accurate and precise measurements [24]. Since accuracy of MRS measurements is very difficult to assess in vivo [24], reproducibility has become the focus of many methodological papers. Assessing the reproducibility (also referred to as repeatability and reliability [25]) is critical in order to enable power calculations and proper study design.

Recent improvements in head coil hardware include an increase in the number of phased-array coils because these have been shown to increase the signal-to-noise (SNR). However, these advances have mostly benefitted measurements in cortical regions; the insula, which is further away from the coils, does not see the same increase in SNR. This may also explain the relatively fewer attempts to measure neurochemistry in this region, relative to the cortical regions, such as the dorsolateral prefrontal cortex (DLPFC) and the anterior cingulate cortex (ACC) [25,26]. In addition, there is a large discrepancy in the voxel sizes and placements in MRS insula studies. Furthermore, there is a lack of reproducibility measurements for the metabolites of interest here (standard five + GABA). The goal of this paper is to assess the reproducibility of the measurements of GABA and other standard metabolite concentrations in the insula. We will measure within session/back-to-back (B2B, on Day1 and Day2, separately) and between session/day-to-day (D2D) reproducibility. GABA will be obtained from the MEGA-PRESS_diff_ spectrum and the other five aforementioned standard metabolites, will be obtained from MEGA-PRESS_editOFF_. As a reference, we will compare the resulting reproducibility metrics to those obtained for a generally accepted short-TE PRESS sequence with all parameters equal other than the TE and the edited pulse. In doing so, we will determine if a short-TE PRESS sequence is necessary for the measurement of any of the five standard metabolites, given a MEGA-PRESS GABA scan is already being performed. This will also provide a reference for the GABA reproducibility metrics.

## 2. Materials and Methods

### 2.1. Participants

Twenty-nine healthy volunteers for MEGA-PRESS scans (age 24 ± 3 years, 20–32 years, 14M/15F) and 17 healthy volunteers for PRESS scans (age 25 ± 4 years, 19–33 years, 7M/10F) were recruited by word-of-mouth as approved by our institution’s Research Ethics Board (REB) for this study. Only healthy subjects, 18 years of age and older, were included if they could keep coffee and alcohol intake and general activities consistent on both scan days. Healthy participants were considered those who self-reported to never being diagnosed with a psychiatric or major neurological illness, including severe learning disabilities and migraines, in addition to never experiencing a major brain trauma (e.g., epilepsy, stroke, seizures). Other exclusion criteria were: metal or electronic implanted devices, severe claustrophobia, and pregnancy. 

### 2.2. Scanning Procedures

MEGA-PRESS and PRESS participants were scanned in separate arms of the study, using the same localization procedure and scanning schedule. All subjects were scanned four times. The first two scans were conducted back-to-back (B2B) in a single session to minimize physiological variability and capture scanner stability. Another set of B2B scans was conducted 1–3 days later at a similar time of the day to capture day-to-day variability in addition to scanner stability across sessions/days (D2D).

**Figure 1:**
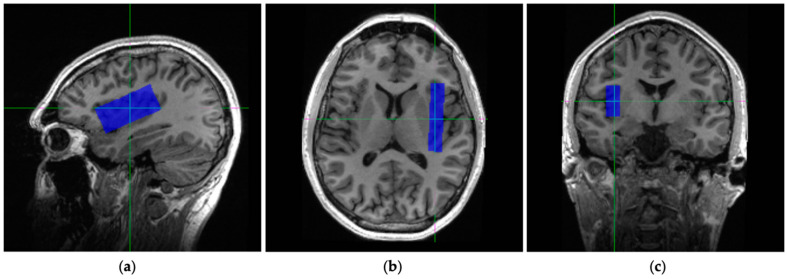
Images illustrating voxel placement of the insula. Voxel is placed 55 mm in anterior-posterior, 12 mm right-left, and 25 mm in superior-inferior direction (with 16.5 mL total volume). The voxel placement is presented in (**a**) sagittal, (**b**) axial, and (**c**) coronal views.

All ^1^H MRS measurements were performed on a 3T, GE MR 750 scanner (General Electric, Waukesha, WI, USA) with a 32-channel head coil (Nova Medical Inc., Wilmington, MA, USA). The scanning protocol included an anatomical T_1_-weighted image using a stock 3D IR-prepared fast spoiled-gradient (FSPGR) sequence (BRAVO, TE = 3.0 ms, TR = 6.7 ms, T1 = 650 ms, flip angle = 8°, resolution = 0.9 mm^3^, scan time = approximately 5 min). T_1_-weighted images were acquired for MRS voxel placement and for grey matter (GM), white matter (WM), and cerebral spinal fluid (CSF) segmentation to enable partial volume correction (see below). Anatomical landmarks were used as a reference to ensure the reproducibility of the voxel placement on the right insula. Initially, axial and coronal images were reformatted from the sagittal BRAVO T_1_-weighted scan. A voxel was then placed on an oblique axial image parallel to lateral/Sylvian fissure, while ensuring that the voxel stayed on the sagittal plane above the temporal lobe. The voxel was kept away from the caudate and temporal gyrus on the axial and coronal plane. The insula voxel extended 12 mm (right-left), 55 mm (anterior-posterior), and 25 mm (superior and inferior), for a total volume of 16.5 mL (Figure 1).

^1^H MRS data was acquired using two different sequences: a single voxel (SV) PRESS (Point Resolved Spectroscopy) sequence [27] and a MEGA-PRESS (Meshcher-Garwood Point Resolved Spectroscopy) [22,28] sequence which uses the J-difference editing technique for the measurement of GABA. J-difference editing involves an interleaved acquisition of spectra with two differing conditions, both with a pair of frequency selective “editing” RF pulses (pulse width = 14.4 ms) applied: edit-ON, where the editing RF pulses placed at 1.9 ppm invert the GABA-H3 spins located at 1.89 ppm (which refocuses the evolution of J-coupled GABA-H4 spins at 3.0 ppm); and edit-OFF where the editing RF pulses are placed at 7.5 ppm, where no metabolite resonances are located (equivalent to having no editing pulse applied). The subtraction of the two conditions results in a difference spectrum where the GABA resonant peak can now be observed, which is otherwise obscured by the larger creatine resonant peak at 3.0 ppm.

Due to its close proximity to the edit-ON RF pulse at 1.9 ppm, glutamate and glutamine (Glx) are also edited alongside GABA (co-editing) and are observed at 3.75 ppm in the difference spectrum. Additionally, macromolecule (MM) resonances reside by 1.9 ppm and are co-edited, resulting in a MM peak at 3.0 ppm. This means that the GABA peak we observe in the difference spectrum is contaminated with MM signal; thus, we will refer to our measurement of GABA as GABA+ (GABA + MM) throughout the rest of this manuscript.

Previous work [29,30] showed that Glx measured from the edit-OFF spectra (MEGA-PRESS_editOFF_) is more reproducible (i.e., lower CV%); thus, the Glx peak in the difference spectra (MEGA-PRESS_diff_), although fit by Gannet, will not be further analyzed here. A CHESS (Chemical Shift Selective Saturation) sequence [31] was applied for optimal water suppression in both cases. The water suppressed data was acquired subsequent to the acquisition of 16 water unsuppressed lines, which are used to perform internal tissue water referencing. Shimming was performed using the manufacturer’s automated shimming routine (AUTOSHIM), to achieve a full-width at half maximum (FWHM) ≤ 12 Hz at the time of scanning. Other scanning parameters are outlined in Table 1.

### 2.3. MRS Data Analysis

The PRESS and MEGA-PRESS_editOFF_ data were analyzed using LCModel (Linear Combination of Model Spectra) software (version 6.3-0E) [32] to obtain concentration values for the five standard metabolites at two different TE values (TE = 35 ms for PRESS and TE = 68 ms for MEGA-PRESS). Data for MEGA-PRESS_editOFF_ spectra were parsed, frequency corrected, and combined using the FID-A toolkit [33] prior to LCModel analysis. For PRESS data, the included LCModel gamma simulated basis set for TE = 35 ms was used for analysis. For the MEGA-PRESS_editOFF_ data, basis spectra were acquired from chemical phantoms for TE = 68 ms.

**Figure 2:**
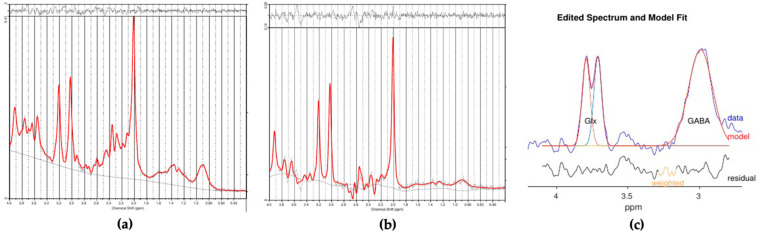
LCModel MRS Spectra from (**a**) PRESS and (**b**) MEGA-PRESS sequence. (**c**) Modeling of GABA signal: GABA-edited spectrum is shown in blue, while the model of best fit is displayed in red. Gannet software uses a simple Gaussian model by default. The black lines demonstrate the residual between the blue and red lines.

GABA+ was fitted and quantified in the difference spectra using Gannet 3.1 [23]. Modifications to GannetFit.m were required to omit the sinusoidal and linear baseline fitting terms, which would occasionally result in obviously underestimated GABA+ and overestimated Glx areas. Gannet and SPM 12 (www.fil.ion.ucl.ac.uk/spm, accessed on 1 June 2021) were used for voxel to T_1_ weighted image registration. Figure 2a–c show the LCModel outputs for PRESS and MEGA-PRESS_editOFF_, and the results generated by Gannet with the fitted Glx and GABA+ peaks, respectively. 

All metabolites are reported in institutional units (IU), where the unsuppressed water signal was used as internal water reference. The results were corrected for water relaxation and density in the tissue compartments [34] using CSF/GM/WM fractions in the voxel, resulting from the tissue segmentation obtained with the “*fast*” algorithm from FSL [35]. The percentage of voxel overlaps for D2D was also determined with FSL. We performed a first quality control step using standard criteria for LCModel data inclusion: SNR > 10, FWHM < 0.1 ppm, CRLB < 15%, and looking for poor quality upon inspection of the PRESS and MEGA-PRESS_editOFF_ spectra given by the LCModel output. GABA+ data was inspected for any outliers (based on standard deviations and the mean across all subjects) of the relevant metrics for data quality/goodness-of-fit output by Gannet: H2O-FWHM, H2O-FitError, GABA-FWHM, and GABA-FitError, as per ref. Kurcyus et al., 2018 [36].

### 2.4. Statistical Analysis

Statistical analysis was performed with SPSS version 25 (IBM, Chicago, IL, USA). Metabolite concentrations were expressed in terms of means and standard deviations. Reproducibility, both back-to-back (B2B) and day-to-day (D2D) were calculated using Pearson’s correlation *r* and its *p*-value by assuming the two-tailed distribution. Coefficients of variations (CV% = (M/SD) × 100%, where M = mean, and SD = standard deviation) were computed to assess repeatability, indicating within-subject variance because the means and standard deviations were for two within-subject test-retest values [25,26]. To identify any biases, we used Bland–Altman plots for our test-retest data for B2B and D2D comparisons [37]. We expect D2D metrics to show poorer reproducibility as the D2D variations from physiological sources and voxel placement are included, in addition to any scanner instabilities (and minimal physiological variations) captured by the B2B metrics. We computed correlations between the D2D voxel overlap and D2D reproducibility metrics to see if voxel placement was driving any of the effects.

In the case of poor B2B reproducibility for the relatively short scan time (<7 min) for GABA+ measurements, we plan to average the two B2B scans to get a result that would be equivalent to that obtained with a scan that is twice as long (2 × 6:46 min = 13.5 min). In such a case, we will assess D2D metrics for the averaged B2B values for all the standard metabolites, as well as GABA+, to make use of all the edit-OFF data acquired for a reproducible GABA+ scan. Averaging would not be performed for the 3.5 min PRESS data which is used as a reference here. Intraclass correlation coefficient (ICC) was used for measuring reliability, depending on both within- and between-subject variance [25]. Using SPSS, single-rating, absolute-agreement for two-way fixed-effects ICC was calculated [38], while assuming the following convention: poor (ICC < 0.4), moderate (0.4 < ICC <0.59), good (0.6 < ICC < 0.74), and excellent (ICC > 0.75) [26].

## 3. Results

### 3.1. Within and between Session Reproducibility

Reproducibility metrics for PRESS and MEGA-PRESS_editOFF_ are shown in Table 2 and Table 3. All data derived from the PRESS scans (*n* = 17) met quality criteria. For MEGA-PRESS_editOFF_, two participant datasets were removed from analysis (*n* = 27) upon screening for excessive movements during the scans, as noted by the MR technologist at the time of scanning and corroborated by the spectral quality check: one dataset had large lipid contamination in the LCModel outputs of the MEGA-PRESS_editOFF_ spectra for both scans on Day2 (likely due to motion between the anatomical scan used for voxel placement and the MRS scan, resulting in poor voxel placement); the second dataset had one scan with excessive head motion resulting in excessive noise visible on one of the four MEGA-PRESS_editOFF_ spectra output by LCModel. Although the latter case reached standard LCModel criteria (SNR = 17, FWHM = 0.086) these values were considered poor for the given scan, particularly the FWHM, where average values of SNR and FWHM were 21.90 and 0.04 ppm, respectively (See Section 3.3). Based on the CRLB% cut-off criteria for the MEGA-PRESS_editOFF_ spectra, two additional data points were removed from Day1 for Glx (*n* = 25) and one from Day2 for each of Ins (*n* = 26) and Glx (*n* = 26), resulting in D2D group sizes of *n* = 26 and *n* = 24 for Ins and Glx, respectively. Interestingly, the poorly fitted Glx were reported for different participants on different days and there was no overlap of subjects with poor fits of the two metabolites, Glx and Ins, so these spurious effects were not subject-dependent. B2B correlation metrics (*r* and ICC) were significant in all cases, across both scans, for the standard metabolites. B2B correlations were only significant for GABA+ measurements on Day2 (more on this in the Discussion section). Percentage changes for these metrics (Day2 relative to Day1) are indicated in Table 2, for all significant cases (all metabolites, except GABA+). In addition, the CV% values shown in Table 2 are not statistically significantly different across days. 

**Figure 3:**
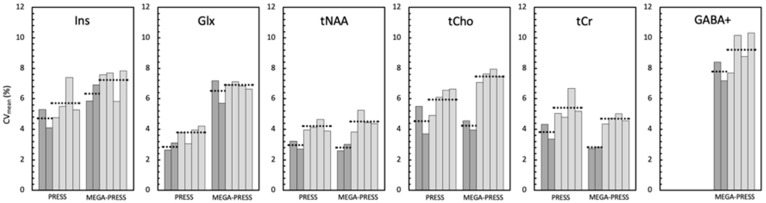
Bar graphs show the CV% of B2B and D2D pairwise comparison across PRESS and MEGA-PRESS for standard metabolites and GABA+. Each bar is an average CV% value across subjects (PRESS *n* = 17; MEGA-PRESS *n* = 25–27). Dark grey bars represent B2B measurements (Day1 and Day2). Light grey bars represent D2D measurements (from left to right, D1S1D2S1; D1S2D2S2; D1S1D2S2; D1S2D2S1) (D is Day, S is Session). Horizontal dotted lines indicate mean of the two B2B CV% and mean of the four D2D CV% for easier visualization. Values are given in Table 2 and Table 3 and Supplementary Tables S1 and S2.

Across session D2D, reproducibility metrics include the following four pair-wise comparisons: (i) D2D-S1, (ii) D2D-S2, (iii) D1S1-D2S2, and (iv) D1S2- D2S1, where D# represents Day#, and S# represents scan # of B2B scans (# = 1,2). Reproducibility for D2D scans are compared across PRESS and MEGA-PRESS in Table 3. Here, we also report the data range from the 4 sets of D2D scan comparisons and the average and standard deviation of the four D2D values of CV% (average is shown by dashed horizontal line in Figure 3). Datapoints that did not have significant correlations (*r* and ICC) were excluded from the reported data range for *r* and ICC. For PRESS, two Ins datasets (D2D-S2, *p* = 0.05; D1S1-D2S2, *p* = 0.3) and one tCr dataset (D1S1-D2S2, *p* = 0.07) were excluded from the *r* and ICC range. For MEGA-PRESS_editOFF_ data, there were significant correlations (*r*) for all 5 standard metabolite measurements obtained using LCModel for D2D scans (*r* = 0.53–0.84; *p* = <0.01). ICC values were also significant in all cases for MEGA-PRESS_editOFF_ data (ICC = 0.51–0.84). For MEGA-PRESS, GABA+ only had one D2D pair with significant *r* and ICC (D2D-S1, *p* = 0.035).

For both PRESS and MEGA-PRESS sequence, B2B CV% are, on average, lower than D2D CV% for all 5 standard metabolites, as indicated by the dashed horizontal lines in Figure 3. While B2B and D2D CV% for MEGA-PRESS were higher than PRESS for Ins, Glx, and tCho, they were comparable. In addition, we noted lower B2B (on Day1 and Day2) and all D2D CV% for tCr in MEGA-PRESS_editOFF_ (B2B: 2.74% and 2.82%; D2D: 4.35%, 4.72%, 5.02%, and 4.55%) compared to PRESS (B2B: 4.33% and 3.35%; D2D: 5.04%, 4.79%, 6.67%, and 5.18%) (Figure 3). MEGA-PRESS_editOFF_ also appears to have higher ICC overall compared to PRESS, with an average of 0.80 across the five standard metabolites for MEGA-PRESS_editOFF_ and 0.70 for PRESS. In addition, no notable biases were identified using BA plots for any of the comparisons (data not shown).

GABA+ measurements were inspected for any outliers (>3 SDs from the mean) of the relevant data quality metrics output by Gannet: H2O-FWHM, H2O-FitError, GABA-FWHM, and GABA-FitError [36]. This resulted in a single outlier for H2O-FWHM and a single outlier for GABA-FitError. However, removal of these outliers did not result in significant changes to reproducibility metrics; thus, these values are not excluded in the presented data (more on this in the Discussion section).

### 3.2. Voxel Overlap and Metabolite Concentration

By co-registering the two T_1_-weighted images with positioned MRS insula voxel on each scan day, we were able to compute the percentage voxel overlap. There was an average %voxel overlap of 82.3% ± 8.9% between Day1 and Day2 scans for PRESS and MEGA-PRESS. The difference between %CSF/GM/WM composition for Day1 and Day2 scans was 0.6%, 0.2%, and 0.8%, respectively (data not shown), indicating there were minimal changes to voxel composition. The degree of voxel overlap also did not correlate with reproducibility values or affect metabolite measurements (*p* > 0.05), suggesting there were no significant changes in the neurochemistry related to voxel placement or CSF/GM/WM composition. This is consistent with what has been observed in other work [39]. The metabolite concentrations reported for TE = 68 ms were lower than those reported for TE = 35 ms, as expected due to the longer TE (Table 4). The reductions were close to 30% for Ins and tCho and closer to 10% for tNAA and tCr. However, the measurement of Glx was drastically reduced by more than 60% (more on this in the Discussion section). We are not reporting the differences in these mean metabolite concentrations across days because we found that these were not statistically significantly different.

### 3.3. FWHM, Signal-to-Noise Ratio, and Tissue Heterogeneity

To evaluate spectral quality and to control for potential group differences, here, we report the FWHM, SNR, CRLB%, and the composition of CSF, GM, and WM across PRESS and MEGA-PRESS. The two groups did not significantly differ in FWHM values nor in the composition of CSF, GM, and WM in the insula voxel (Table 5). Both groups reported good spectral quality overall. The CRLB% for the standard metabolites are also comparable between PRESS and MEGA-PRESS_editOFF_ for tNAA, tCho, and tCr (which are all very low, <3.0). However, the CRLB% are almost double for Glx and Ins for MEGA-PRESS_editOFF_ versus PRESS data but still well within the cut-off value of 15%, on average.

### 3.4. Sex Differences

Sex differences in metabolite concentration have previously been reported. For instance, GABA+ and Glx concentration were found to be significantly higher in males dorsolateral prefrontal cortex [40], whereas, in the anterior cingulate cortex, females had higher levels of tCr, NAA, and glutathione and lower levels of glutamine [41,42]. To date, no sex dependent differences in metabolite concentrations have been reported in the insula [43]. We investigated whether there were sex differences between metabolite concentration in the insula using data from MEGA-PRESS_editOFF_. Overall, we detected no significant metabolite variations between the male (*n* = 12) and female (*n* = 15) groups (all *p* > 0.05; Supplementary Table S3).

## 4. Discussion

This study has been designed to assess reproducibility of metabolite concentration measurements using a MEGA-PRESS scan with parameters optimized for the measurement of GABA+ in a 16 mL long and narrow voxel in the insula. We measured reproducibility both within session (B2B) and across sessions that were 1–3 days apart (D2D), with the assumption that the B2B reproducibility metrics (CV%, *r*, and ICC) would capture scanner-based instabilities with minimal physiological changes. Therefore, the B2B values are used as a reference to determine how much day-to-day physiological variations and possible inconsistencies in voxel placement affect the reproducibility of our measurements. This is helpful because, although there is some consensus in the literature to use these metrics to measure reproducibility, there is still no gold standard for what constitutes a good value for CV. This is in contrast to Pearson’s *r* and ICC which, when significant (*p* < 0.05), can be associated with levels of correlation strength (e.g., *r* > 0.7 is considered a strong correlation, and ICC > 0.75 is considered excellent reliability). Here, we use Pearson’s *r* to assess if the test-retest values are meaningful. Because ICC is dependent on the intra- and inter-subject variability, it reflects the measurement variability in relation to the variability in the cohort being sampled. Given that our groups are healthy and within a fairly tight age range (most are in their 20s, with only 2 subjects > 30 years), we can interpret high ICC values (>0.7) to reflect highly reproducible measurements. If we get moderate to good correlations, we can look at CV% across sessions in relation to the CV% within session. It is well-appreciated that the value will be dependent on voxel size, location, and scanning parameters, such as TE and TR, since these values will not only affect the amount of noise in the measurement but also the mean metabolite concentrations, which are used to compute CV%. This is also why we expect to obtain varying CV% for the different metabolites, which are at very different concentrations. With the addition of the high quality short-TE PRESS scan in the same voxel, and also sampled within and across sessions, the design of this study provides a reference value for the CV% for D2D reproducibility.

### 4.1. Mean Concentration Values and CRLB

The average concentrations (in IU) for the five standard metabolites and GABA+ are given in Table 4, and average CRLB% (LCModel output) is given in Table 5. Given that CRLB are shown as a relative amount (% relative to the average metabolite concentration), we can compute approximate absolute CRLB values (CRLB_abs_) as: CRLB_abs_ = CRLB% × average (concentration). Using this on all average values reported in Table 4 and Table 5 and comparing results for PRESS/MEGA-PRESS data gives: CRLB_abs_ = 0.36/0.58 for Ins, 1.02/0.79 for Glx, 0.33/0.30 for tNAA, 0.07/0.08 for tCho, and 0.23/0.21 for tCr. Thus, we can see that only in the case of Ins, MEGA-PRESS gives a larger absolute error/uncertainty in the fit than PRESS. This may explain the one MEGA-PRESS_editOFF_ spectrum that did not meet the cut-off criterion (CRLB% > 15%) for Ins. In fact, for Glx, the error/uncertainty is lower for MEGA-PRESS than PRESS, and it would appear that the exclusion criteria (CRLB% < 15% which led to the exclusion of three data points) may have been too restrictive in this case as the higher CRLB% values appear to be due to low concentrations of Glx rather than a poor fit. 

Although T2 is expected to vary across metabolites (see values given in Table 1 of Dhamala et al., 2019 [24]) and regions [44], with shortest value expected for tCr (e.g., 158 ms in cortical GM) and the longest value expected for tNAA (e.g., 288 ms in cortical GM), the relative amount of metabolites, across the TE values used here, is not expected to vary drastically. Given the values in Table 4, we see similar ratios of metabolite concentrations across our 2 sequences (across our 2 different groups): tNAA/tCr = 1.42 for both MEGA-PRESS and PRESS, tCho/tCr = 0.26 and 0.31 for MEGA-PRESS and PRESS, respectively, and Ins/tCr = 0.76 and 0.61 for MEGA-PRESS and PRESS, respectively. However, the value for Glx/tCr changes drastically, from 2.19 for PRESS to 0.94 for MEGA-PRESS. This inconsistency in the relative amount of Glx across TE values has been noted previously [26,29,45], where both PRESS and MEGA-PRESS scans, with similar scanning parameters to those used in this study, were performed on the same subjects, back-to-back. In fact, these studies found a lack of correlation for the Glx values measured using the different sequences and the Glx measurement derived from the MEGA-PRESS_editOFF_ spectra resulted in approximately half the amount of Glx concentration as that derived for PRESS (we observe an even larger reduction of 9.85/25.42 = 0.39). This lack of concordance across sequences has been noted as “surprising”, and, despite some efforts to explain possible sources for these differences (e.g., contributions from macromolecules at lower TE or effects of the editing pulse on the Glx to macromolecule ratio [29,45]), this discrepancy remains poorly understood. Furthermore, in these studies, the Glx measurement taken from the MEGA-PRESS_diff_ spectra appears to be closer to the PRESS measurement (i.e., less reduced), but the values are still not correlated across sequences. Although we cannot compute correlations across scans because we did not scan the same subjects with PRESS and MEGA-PRESS, the large change in Glx concentration at the longer TE is very relevant to this study: It is an important source of the reduction in the reproducibility values for Glx we see when comparing measurements from MEGA-PRESS to those from PRESS.

### 4.2. B2B Reproducibility

We obtained significant Pearson’s correlation *r*-values and ICC for all our PRESS B2B comparisons, with strong correlations (*r* > 0.7 and ICC > 0.75) for all metabolites, except one instance (Day1) for Ins (*r* = 0.59, ICC = 0.57) and one instance (Day1) for tCho (*r* = 0.64, ICC = 0.60). The CV% values ranged from 2.63% to 5.30%, with lowest values for Glx and tNAA (metabolites in highest concentrations) and highest values for Ins and tCho (metabolites in lowest concentrations). We also obtained significant Pearson’s correlation *r*-values and ICC for all our MEGA-PRESS_editoFF_ B2B comparisons, with strong correlations for all metabolites, except one instance (Day1) for Glx (*r* = 0.66, ICC = 0.64) and one instance (Day2) for Ins (*r* = 0.70, ICC = 0.70). The *r* and ICC values tended to be higher in the MEGA-PRESS_editOFF_ dataset, compared to PRESS, and the percentage difference in these metrics across days was lower for MEGA-PRESS_editOFF_ (0–17%) than for PRESS (4–34%), which indicates better stability in the single measurements for MEGA-PRESS_editOFF_. For MEGA-PRESS_editOFF_ B2B data, the CV% values ranged from 2.59% to 7.19%, with the values ranked in increasing order for tNAA, tCr, tCho, and Ins, the same as for PRESS. The only change was that Glx had highest CV% for MEGA-PRESS_editOFF_, whereas it had lowest CV% for PRESS, but this is likely just a reflection of the marked decrease in concentration of Glx for the longer TE of MEGA-PRESS_editOFF_.

**Figure 4:**
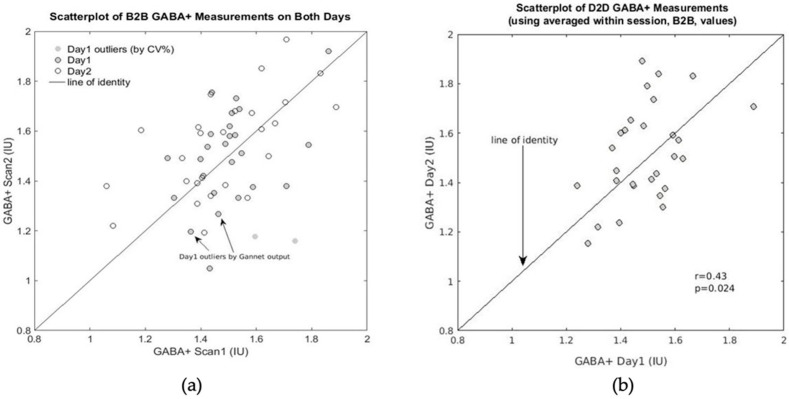
(**a**) GABA+ concentrations (in IU) for B2B data plotted as Scan2 versus Scan1 on both days. Using all data (*n* = 27) on Day2 gives a very significant correlation (*r* = 0.65, *p* = 0.003). Using all data (*n* = 27) on Day1 gives an insignificant correlation (*r* = 0.20, *p* = 0.31), but, if we remove the 2 outliers identified based on large CV% values, we get a significant, albeit low, correlation (*r* = 0.41, *p* = 0.04) for Day1 (*n* = 25). The 2 data points indicated by the arrows were identified as outliers for exceeding 3 × SDs away from the mean values of H2O-FWHM and GABA+-FitError (Gannet output). Removal of these did not improve the significance of the correlation. (**b**) D2D correlations of GABA+ concentrations when B2B measurements are averaged (*r* = 0.43, *p* = 0.024), plotted as Day2 (average of Day2 within session values) versus Day1 (average of Day1 within session values).

For GABA+, we only obtained significant Pearson’s *r* and ICC values for the B2B scans on Day2 with moderately high values (*r* > 0.6 and ICC > 0.6). The poor B2B correlations for Day1 could not be explained as driven by poor GABA+ fitting/data quality, identified by a quality check performed on the Gannet output [36]. There were only 2 GABA+ data points that exceeded the thresholds obtained this way, and elimination of the outlier on Day1 did not improve our B2B correlations for Day1 (still non-significant: *r* = 0.15, *p* = 0.46). In fact, closer observation showed that the CV% for the corresponding subject on Day1 was 10.17%, and it was not responsible for the lack of significant correlation. Based on the CV% values per subject, we were able to identify two subjects with CV% = 28.24% and CV% = 21.51% on Day1 that were driving the lack of correlation; when these two subjects were removed from the GABA+ dataset, a significant B2B correlation for *n* = 25 ((*r*,*p*) = (0.41, 0.04)) was obtained; see Figure 4a.. We also looked at all the data pooled (for both days) because, if we assume that B2B measurements exclusively capture scanner instabilities, the data on different days could be considered independent. This resulted in significant B2B correlations for *n* = 54 (2 × 27), albeit with low strength: *r* = 0.45, *p* = 0.001(see Figure 4a). 

The B2B data for GABA+ can, therefore, be considered too noisy to produce reproducible GABA+ measurements, suggesting that a scan time longer than 6:46 min is necessary. It is important to note that, although *r* and ICC values were non-significant for Day1, the CV% was 8.41%, which is only slightly higher than that obtained for Day2 (7.81%), indicating that CV% alone cannot identify the level of reproducibility of the data. The poor B2B reproducibility measured for GABA+ on Day1 was found to be driven by two subjects with extremely large values for CV% relative to the others (also identified on Bland–Altman plots). This outlier identification is not ideal for two reasons: (i) it requires B2B measurements to compute CV% and these are not usually available, and (ii) it cannot identify which of the two B2B scans is problematic. The fact that the GABA+ quality check method was not able to identify any of the 4 spectra associated with these two problematic data points, demonstrates a lack of adequate measures to assess the quality of GABA+ results on a single spectrum basis. This highlights the need to develop better quality check criteria for the evaluation of GABA+ results from Gannet in the future.

### 4.3. D2D Reproducibility

All D2D correlations (*r* and ICC) were significant for PRESS data, except for a single case for tCr and two cases for Ins. Significant *r* and ICC values were in the moderate range for most metabolites, with the exception of Glx, which had ICC in the good to excellent range. For MEGA-PRESS_editOFF_ data, all D2D paired scans were significantly correlated with *r* and ICC values in the moderate to excellent range, with stronger correlations for tNAA and tCr and lower correlations for Glx and tCho (related to their concentrations). GABA+, D2D correlations were only significant in one case out of the four. This is not surprising given the poor B2B correlations.

In terms of CV%, data are shown in Figure 3 as bar graphs since there was some variation in the values that depended on which pair of scans was used to compute the D2D metric. In all cases, the CV% increased from B2B to D2D values, as can be appreciated by looking at the dashed lines in Figure 3. This consistent step increase from B2B to D2D CV% is expected because the D2D repeatability will be equal to the B2B repeatability, at best, given that it includes additional day-to-day physiological and voxel placement sources of instability. In general, the CV% followed the metabolite concentrations as expected (given that it is a relative measure) with lower values for: tNAA, tCr, and Glx for PRESS, and tNAA and tCr for MEGA-PRESS_editOFF_. In addition, PRESS and MEGA-PRESS_editOFF_ data had comparable CV% for all metabolites except Glx, which was much lower for PRESS (given the change in concentration). GABA+ had CV% values that are higher than any of the PRESS and MEGA-PRESS_editOFF_ data, indicative of poor reproducibility for a 6:46 min scan time.

Due to the poor correlations for the B2B and D2D scans for GABA+ and higher CV% values, it can be concluded that a longer scan time than 6:46 min is required for reproducible GABA+ measurements. Consequently, we computed the average metabolite concentration within session (average of B2B scan results for each subject) of all metabolites obtained using the MEGA-PRESS sequence. The average within session values required a scan time of 13.5 min (shown in Table 6). D2D correlation for averaged within session GABA+ concentration is shown in Figure 4b (*r* = 0.43, *p* = 0.024). Averaged within session scans resulted in significant D2D correlations (*r* and ICC) for all metabolites, with good to excellent correlations for all metabolites, except for tCho, which was moderate (*r* = 0.58, ICC = 0.58), and GABA+ which was poor (*r* = 0.43, ICC = 0.41). The D2D CV% values obtained from the averaged within session values were lower than the average D2D values (listed in Table 3 and shown by the horizontal dashed lines in Figure 3), and they fall within the range of values observed for PRESS data for each metabolite (except Glx, due to the large decrease in concentration). Using the average within session GABA+ measurements, we had a D2D CV% of 7.15%, which is lower than the B2B CV% values obtained for GABA+ on either day. This D2D CV% is also within the range of D2D CV% measured with PRESS for Ins (see Table 3). This confirms that a scan time of 13.5 min for MEGA-PRESS allows for reproducible measures of GABA+ and the other five standard metabolites.

### 4.4. Limitations

Several limitations can be identified with this study. First, the sample sizes across the two cohorts (MEGA-PRESS and PRESS) were not matched for practical reasons, favoring more subjects scanned with the less standard sequence (MEGA-PRESS). We do not expect this to have a significant effect on our results due to the fact that computing r-adjusted values (see Baeshen et al. [26]) for *r* in the range 0.4–0.8 for *n* = 17 and *n* = 29 gives a difference in *r* that is less than 1%. Given the close agreement in reproducibility metrics across PRESS and MEGA-PRESS for the standard metabolites, this is not likely an issue here.

A technical limitation of this study is that different basis sets were used by the LCModel for the different TEs. The TE = 68 ms basis set used has missing signatures with respect to the TE = 35 ms basis set: tCh does not include PCh, just GPC, and tNAA does not include NAAG (just NAA). That does not seem to be a problem since the LCModel did not detect those signatures in the short-TE PRESS spectra (i.e., PCh and NAAG were rarely fit with any non-zero/negligible values). The more notable difference is that the TE = 68 ms basis set did not have PCr (only Cr) since both PCr and Cr are detected by LCModel in the short-TE PRESS spectra, in general. However, the signatures of these two metabolites consist of a single large peak for each, and these peaks largely overlap the same ppm range. Thus, they are likely being absorbed into a single peak for the MEGA-PRESS_editOFF_ fits without any loss of concentration. This is corroborated by noting that tNAA/tCr and tCho/tCr are comparable across scans (PRESS versus MEGA-PRESS) despite the difference in TE values across scans, T2 values across metabolites [24], and basis sets.

Another limitation of this study is that we only looked at Glx from the MEGA-PRESS_editOFF_ spectra due to the expectation that this would provide a more reproducible measurement [30]. However, Glx is also automatically fit by Gannet when fitting GABA+ in the difference spectra (see Figure 2c); thus, future work should involve investigating the resulting Glx values and comparing them with those reported here. 

One main limitation is that we only examined a fixed scan time for GABA+ of 6:46 min. This scan time was chosen because we determined that it produced data with sufficient quality to provide decent fits to the difference spectra with Gannet. Given the voxel size of 16.5 mL and the fact that the insula is not as close to the coils, we expected that reproducible data would necessitate a longer scan time for this voxel than for voxels in cortical regions: anterior cingulate cortex (ACC) and dorsolateral prefrontal cortex (DLPFC), which have been investigated more often. Those usually require a minimum of 10 min scan time. By using two B2B scans of 6:46 min, we tested the ability to obtain good B2B results from this short scan time, with the option of averaging two within session results [26], to improve D2D reproducibility. Although our results show that the within session averaging produced good D2D GABA+ reproducibility, otherwise not observed with the 6:46-min scan time, reproducible D2D scans may be obtained with a shorter scan time (somewhere between 6:46 min and 13.5 min). Thus, the focus of our future investigations will be to gradually merge transients to produce spectra equivalent to scanning from 6:46 min to 13.5 min, as proposed by Brix et al. [46]. The process of merging transients will be conducted for all MEGA-PRESS scans to optimize the reproducibility of all metabolites examined. In addition, we expect that, by merging transients to obtain a single spectrum to be fit once, we will improve upon the values reported here, where a simple averaging of B2B fitting results was performed.

## 5. Conclusions

We have shown that, by using the MEGA-PRESS protocol requiring 13.5 min of scan time, we obtain D2D reproducibility metrics for GABA+ that are comparable to the reproducibility metrics we get for standard metabolites with a 3.5-min short-TE PRESS scan. We have shown that tNAA and tCr, which have the highest concentrations, have equal or better reproducibility when extracting the D2D metrics from the within session average values. For tCho and Ins, with lower concentrations but peaked signatures, the D2D reproducibility values are comparable to those for the short-TE PRESS scan, albeit with an increase in absolute CRLB for Ins. For Glx, there is a large loss in relative concentration for TE = 68 ms versus TE = 35 ms that warrants further investigation. The change in Glx concentration results in higher CV% and CRLB% for MEGA-PRESS than for PRESS (although CRLB_abs_ was found to be lower for MEGA-PRESS). Nonetheless, D2D reproducibility values for Glx obtained from averaging the within session results from MEGA-PRESS_editOFF_ fits are comparable to those obtained for other metabolites (e.g., Ins) with a short-TE PRESS scan. In short, an additional PRESS scan is not necessary, for reproducible data, if a 13.5 min MEGA-PRESS protocol is being used in the insula. However, the Glx values should be taken with caution as the discrepancy in Glx measurements across methods (short-TE PRESS, MEGA-PRESS_editOFF_, MEGA-PRESS_diff_) remains poorly understood [29,45]. Our data has also shown that voxel placement is not a critical factor in obtaining highly reproducible data, and we were not able to detect any sex differences of any of the metabolites in the insula of our young and healthy cohort.

## Figures and Tables

**Table 1:** PRESS and MEGA-PRESS sequence parameters. For MEGA-PRESS, there were 128 acquisitions with editON and 128 acquisitions with editOFF. Both sequences had 16 water unsuppressed acquisitions at the very beginning as is standard for PRESS on a GE scanner.

Scan Parameters	PRESS	MEGA-PRESS
Echo Time (TE)	35 ms	68 ms
Repetition time (TR)	1500 ms	1500 ms
Number of Acquisitions	128	256
Number if Excitations (NEX)	8	8
Number of Points	4096	4096
Spectral Width	5000 Hz	5000 Hz
Scan Time	3 min 36 s	6 min 46 s

**Table 2:** B2B reproducibility metrics comparison: PRESS data (left white column) and MEGA-PRESS data (right grey column). CV% are quoted as mean (M) and standard deviations (SD) across all subjects. Large SD values result due to some very large outliers for CV%. Pearson’s correlation, *r*, and ICC values are denoted with */**, depending on the level of significance. Percentage changes (Day2 relative to Day1) for significant *r* and ICC values are shown in the square brackets.

B2B	Metabolite	*n*		*r* [% Difference from Day1]		CV% M (SD)		ICC [% Difference from Day1]	
Day1	Ins	17	27	0.59 *	0.83 **	5.30 (4.98)	5.85 (3.60)	0.57 **	0.84 **
	Glx	17	25	0.88 **	0.66 **	2.63 (1.87)	7.19 (4.29)	0.87 **	0.64 **
	tNAA	17	27	0.71 **	0.93 **	3.23 (2.48)	2.59 (2.21)	0.69 **	0.91 **
	tCho	17	27	0.64 **	0.91 **	5.50 (6.00)	4.55 (3.79)	0.60 **	0.87 **
	tCr	17	27	0.74 **	0.95 **	4.33 (4.03)	2.74 (2.50)	0.70 **	0.93 **
	GABA+	--	27	--	0.20	--	8.41 (6.84)	--	0.19
Day2	Ins	17	26	0.79 ** [↑ 34%]	0.70 ** [↓ 16%]	4.10 (2.21)	6.92 (5.55)	0.74 ** [↑ 29%]	0.70 ** [↓ 17%]
	Glx	17	24	0.73 ** [↓ 17%]	0.79 ** [↑ 17%]	3.09 (2.25)	5.70 (3.90)	0.81 ** [↓ 7%]	0.74 ** [↑ 16%]
	tNAA	17	27	0.87 ** [↑ 23%]	0.89 ** [↓ 17%]	2.72 (1.85)	3.01 (2.30)	0.84 ** [↑ 22%]	0.88 ** [↓ 3%]
	tCho	17	27	0.80 ** [↑ 25%]	0.91 ** [↑ 0%]	3.70 (2.95)	3.95 (4.31)	0.74 ** [↑ 23%]	0.89 ** [↑ 2%]
	tCr	17	27	0.83 ** [↑ 12%]	0.94 ** [↓ 1%]	3.35 (1.98)	2.82 (2.95)	0.73 ** [↑ 4%]	0.92 ** [↓ 1%]
	GABA+	--	27	--	0.65 **	--	7.18 (5.47)	--	0.62 **

* Correlation is significant at the 0.05 level (2-tailed). ** Correlation is significant at the 0.01 level (2-tailed).

**Table 3:** PRESS and MEGA-PRESS D2D reproducibility metrics comparison. CV% includes mean (SD) and range of the four sets of D2D comparisons (D2D-S1; D2D-S2; D1S1D2S2; D1S2D2S1). The Min and Max *r* and ICC values, for the four sets of D2D comparisons, are reported, and their significance is given by the */** level. Only *r* and ICC values that are significant are included in the reported ranges.

D2D	Metabolite	*n*	CV%		*r*		ICC	
			Mean (SD)	Range	Min.	Max.	Min.	Max
PRESS	Ins	17	5.73 (1.15)	4.76–7.39	0.48 *	0.62 **	0.46 *	0.62 **
	Glx	17	3.75 (0.49)	3.06–4.20	0.66 **	0.79 **	0.66 **	0.79 **
	tNAA	17	4.16 (0.34)	3.90–4.65	0.52 *	0.60 *	0.51 *	0.61 *
	tCho	17	6.05 (0.80)	4.91–6.63	0.55 *	0.75 **	0.46 *	0.70 **
	tCr	17	5.42 (0.85)	4.8–5.18	0.56 *	0.64 **	0.47 *	0.59 *
MEGA-PRESS	Ins	26	7.24 (0.94)	5.83–7.84	0.58 *	0.73 **	0.58 *	0.74 **
	Glx	24	6.87 (0.20)	6.63–7.12	0.54 *	0.71 **	0.55 *	0.67 **
	tNAA	27	4.47 (0.60)	3.81–5.26	0.64 **	0.74 **	0.65 *	0.75 **
	tCho	27	7.52 (0.37)	7.08–7.96	0.53 *	0.58 **	0.51 *	0.58 *
	tCr	27	4.66 (0.28)	4.35–5.02	0.81 **	0.84 **	0.77 **	0.84 **
	GABA+	27	9.24 (1.24)	7.69–10.32	0.41 *	0.41 *	0.38	0.38

* Correlation is significant at the 0.05 level (2-tailed). ** Correlation is significant at the 0.01 level (2-tailed).

**Table 4:** Means (standard deviations) of metabolite concentration (IU) in the insula with PRESS and MEGA-PRESS, averaged across B2B values for each scan day.

	Metabolite	PRESS	MEGA-PRESS
Day1 Average	Ins	8.88 (0.91)	6.42 (1.00)
	Glx	25.42 (2.09)	9.85 (1.29)
	tNAA	16.52 (1.10)	14.87 (1.61)
	tCho	3.58 (0.42)	2.70 (0.40)
	tCr	11.60 (1.17)	10.50 (1.35)
	GABA+	--	1.48 (0.09)
Day2 Average	Ins	8.96 (0.74)	6.39 (0.94)
	Glx	25.79 (2.00)	9.85 (1.22)
	tNAA	16.70 (1.27)	15.06 (1.56)
	tCho	3.52 (0.29)	2.71 (0.46)
	tCr	11.64 (0.81)	10.59 (1.46)
	GABA+	--	1.50 (0.20)

**Table 5:** Data quality values ((means (standard deviations)): Full-width at half maximum (FWHM), signal-to-noise ratios (SNR), CRLB% for the five metabolites, and voxel composition (fractions of CSF/GM/WM adding up to 1). For GM/WM/CSF, average fractions across days were taken for obtaining M(SD).

	FWHM (ppm)	SNR	CRLB%					CSF	GM	WM
			Ins	Glx	tNAA	tCho	tCr			
PRESS (*n* = 17)	0.05 (0.01)	34.29 (3.04)	4.0 (0.4)	4.0 (0.4)	2.0 (0.0)	2.0 (0.0)	2.0 (0.3)	0.17 (0.03)	0.63 (0.03)	0.19 (0.03)
MEGA-PRESS (*n* = 27)	0.04 (0.01)	21.90 (5.66)	9.0 (1.3)	8.0 (1.7)	2.0 (0.3)	3.0 (0.3)	2.0 (0.4)	0.18 (0.03)	0.63 (0.04)	0.18 (0.03)

**Table 6:** Reproducibility metrics obtained for D2D when the average B2B values are used; thus, these are values for an equivalent of 13.5 min scan time. CV is given as M(SD)) with values computed across subjects. Correlation metrics, *r*, and ICC, are given, with their significance indicated by the */**.

MEGA-PRESS						
	Metabolite	*n*	*r*	*p* (2-Tailed)	CV% M (SD)	ICC
D2D 13.5 min	Ins	26	0.74 **	<0.001	6.13 (4.24)	0.75 **
	Glx	24	0.74 **	<0.001	5.06 (3.75)	0.74 **
	tNAA	27	0.74 **	<0.001	4.09 (3.21)	0.74 **
	tCho	27	0.58 **	0.002	6.64 (6.68)	0.58 **
	tCr	27	0.84 **	<0.001	4.03 (3.07)	0.85 **
	GABA Ave	27	0.43 *	0.024	7.15 (4.03)	0.41 *

* Correlation is significant at the 0.05 level (2-tailed). ** Correlation is significant at the 0.01 level (2-tailed).

## Supplementary Materials

The following are available online at https://www.mdpi.com/article/10.3390/brainsci11111538/s1, Table S1: MEGA-PRESS_editOFF_ B2B and D2D Reproducibility Data, Table S2: PRESS B2B and D2D Reproducibility Data, Table S3: Metabolite concentrations compared between male and female participants using Day2 scan averages. The concentrations were derived from MEGA-PRESS edit OFF scans. M = mean and SD = standard deviation are computed across subjects.
