# Supplementary material for: MR Spectroscopy of the Insula: Within- and between-Session Reproducibility of MEGA-PRESS Measurements of GABA+ and Other Metabolites
Source: Brain Sci. 2021 Nov 19;11(11):1538. doi: 10.3390/brainsci11111538 (PMC8615582; doi:10.3390/brainsci11111538)
Supplement: Supplementary file 1 [file brainsci-11-01538-s001.zip › brainsci-1421721-supplementary.pdf]

MR Spectroscopy of the Insula: Within- and Between-Session Reproducibility of MEGA-PRESS measurements of GABA+ and other metabolites

Shyu, C.; Elsaid, S.; Truong, P.; Chavez, S.; Le Foll, B.

Supplementary Materials

			MEGA-PRESS			
	Metabolite	n	r	P (2-tailed)	CV%	ICC
Day 1 B2B	GABA	27	0.20	0.308	8.41 (6.84)	0.19
	Ins	27	0.83	<0.001	5.85 (3.60)	0.84
	Glx	25	0.66	<0.001	7.19 (4.29)	0.64
	tNAA	27	0.93	<0.001	2.59 (2.21)	0.91
	tCho	27	0.91	<0.001	4.55 (3.79)	0.87
	tCr	27	0.95	<0.001	2.74 (2.50)	0.93
Day 2 B2B	GABA	27	0.65	<0.001	7.18 (5.47)	0.62
	Ins	26	0.70	<0.001	6.92 (5.55)	0.70
	Glx	26	0.79	<0.001	5.70 (3.90)	0.74
	tNAA	27	0.89	<0.001	3.01 (2.30)	0.88
	tCho	27	0.91	<0.001	3.95 (4.31)	0.89
	tCr	27	0.94	<0.001	2.82 (2.95)	0.92
D2D Avg	GABA Ave	27	0.43	0.024	7.15 (4.03)	0.41
	Ins	26	0.74	<0.001	6.13 (4.24)	0.75
	Glx	24	0.74	<0.001	5.06 (3.75)	0.74
	tNAA	27	0.74	<0.001	4.09 (3.21)	0.74
	tCho	27	0.58	0.002	6.64 (6.68)	0.58
	tCr	27	0.84	<0.001	4.03 (3.07)	0.85
D2D-S1	GABA	27	0.41	0.035	7.69 (5.07)	0.38
	Ins	26	0.67	<0.001	7.59 (5.26)	0.68
	Glx	24	0.54	0.006	6.91 (5.08)	0.55
	tNAA	27	0.74	<0.001	3.81 (3.30)	0.74
	tCho	27	0.53	0.005	7.08 (6.84)	0.53
	tCr	27	0.82	<0.001	4.35 (3.14)	0.81
D2D-S2	GABA	27	0.38	0.053	10.17 (5.43)	0.35
	Ins	26	0.64	<0.001	7.69 (5.42)	0.65
	Glx	24	0.65	0.001	7.12 (4.77)	0.64
	tNAA	27	0.64	<0.001	5.26 (3.63)	0.67
	tCho	27	0.58	0.002	7.63 (6.98)	0.58
	tCr	27	0.82	<0.001	4.72 (3.89)	0.82
D1S1D2S2	GABA	27	0.13	0.515	8.79 (6.31)	0.12
	Ins	26	0.73	<0.001	5.83 (6.18)	0.74
	Glx	24	0.64	0.001	6.83 (4.52)	0.62
	tNAA	27	0.66	<0.001	4.45 (4.14)	0.65
	tCho	27	0.53	0.005	7.96 (6.96)	0.51
	tCr	27	0.81	<0.001	5.02 (3.34)	0.77
D1S2D2S1	GABA	27	0.29	0.138	10.32 (5.31)	0.30
	Ins	26	0.58	0.002	7.84 (6.29)	0.58
	Glx	24	0.71	<0.001	6.63 (4.42)	0.67
	tNAA	27	0.74	<0.001	4.37 (3.42)	0.75
	tCho	27	0.57	0.002	7.43 (7.26)	0.57
	tCr	27	0.84	<0.001	4.55 (3.11)	0.84

*. Correlation is significant at the 0.05 level (2-tailed).
**. Correlation is significant at the 0.01 level (2-tailed).

Supplementary Table S1. MEGA-PRESS$_{editOFF}$ B2B and D2D Reproducibility Data.

PRESS						
	**Metabolite**	***n***	***r***	***P* (2-tailed)**	**CV%**	**ICC**
Day 1 B2B	Ins	17	0.59	0.01	5.30 (4.98)	0.57
	Glx		0.88	<0.001	2.63 (1.87)	0.87
	tNAA		0.71	0.001	3.23 (2.48)	0.69
	tCho		0.64	0.006	5.50 (6.00)	0.60
	tCr		0.74	<0.001	4.33 (4.03)	0.70
Day 2 B2B	Ins	17	0.79	<0.001	4.10 (2.21)	0.74
	Glx		0.73	<0.001	3.09 (2.25)	0.81
	tNAA		0.87	<0.001	2.72 (1.85)	0.84
	tCho		0.80	<0.001	3.70 (2.95)	0.74
	tCr		0.83	<0.001	3.35 (1.98)	0.73
D2D-S1	Ins	17	0.61	0.009	4.76 (3.97)	0.62
	Glx		0.66	0.004	3.77 (3.35)	0.67
	tNAA		0.60	0.01	3.96 (2.96)	0.61
	tCho		0.75	<0.001	4.91 (3.23)	0.70
	tCr		0.64	0.006	5.04 (3.03)	0.58
D2D-S2	Ins	17	0.48	0.05	5.51 (4.90)	0.46
	Glx		0.79	<0.001	3.06 (2.22)	0.79
	tNAA		0.52	0.03	4.13 (3.24)	0.51
	tCho		0.65	0.005	6.10 (3.08)	0.59
	tCr		0.60	0.01	4.79 (3.49)	0.59
D1S1D2S2	Ins	17	0.25	0.34	7.39 (4.49)	0.22
	Glx		0.69	0.002	3.96 (3.07)	0.66
	tNAA		0.54	0.03	4.65 (3.67)	0.52
	tCho		0.55	0.02	6.56 (4.33)	0.51
	tCr		0.45	0.07	6.67 (3.63)	0.40
D1S2D2S1	Ins	17	0.62	0.008	5.27 (4.56)	0.60
	Glx		0.68	0.003	4.20 (2.62)	0.69
	tNAA		0.56	0.02	3.90 (2.66)	0.57
	tCho		0.56	0.02	6.63 (5.33)	0.46
	tCr		0.56	0.02	5.18 (3.90)	0.47

*. Correlation is significant at the 0.05 level (2-tailed).
**. Correlation is significant at the 0.01 level (2-tailed).

Supplementary Table S2. PRESS B2B and D2D Reproducibility Data.

**Metabolite Concentrations (IU)**						
	**Females**		**Males**		**Statistical Values**	
Metabolite	n	Day 2 ave (M ± SD)	n	Day 2 ave (M ± SD)	Independent Sample t-test*	p-value (2-tailed)
GABA	15	1.54 ± 0.17	12	1.52 ± 0.22	t(27) = -0.2	0.82
Ins	15	6.25 ± 0.85	11	6.58 ± 1.64	t(26) = 0.85	0.41
Glx	13	9.88 ± 1.29	11	9.82 ± 1.20	t(24) = -0.11	0.91
tNAA	15	14.98 ± 1.26	12	15.15 ± 1.92	t(27) = 0.26	0.80
tCho	15	2.70 ± 0.43	12	2.72 ± 0.50	t(27) = 0.14	0.89
tCr	15	10.49 ± 1.34	12	10.72 ± 1.64	t(27) = 0.39	0.70

Supplementary Table S3. Metabolite concentrations compared between male and female participants using Day2 scan averages. The concentrations were derived from MEGA-PRESS edit OFF scans. M=mean and SD=standard deviation are computed across subjects.
